# Special Issue: Emergency Medicine: Clinical Advances and Challenges in Diagnosis and Treatment

**DOI:** 10.3390/jpm14030263

**Published:** 2024-02-28

**Authors:** Ovidiu Alexandru Mederle, Popa Daian Ionel, Williams Gabriela Carmen

**Affiliations:** 1 Surgery Department, Faculty of Medicine, “Victor Babes” University of Medicine and Pharmacy, E. Murgu Square No. 2, 300041 Timisoara, Romania; mederle.ovidiu@umft.ro; 2 Emergency Department, Emergency Clinical Municipal Hospital, Gheorghe Dima Street, Number 5, 300079 Timisoara, Romania; drcarmen.williams@yahoo.com

## 1. Introduction

The development of Emergency Medicine brings various challenges. However, this journey offers many opportunities, and with the joint efforts and hard work of medical professionals, continuously updated medical technology and concepts, Emergency Medicine will undergo more historic advancements. The landscape of Emergency Medicine is characterized by its continuous evolution. It has significantly transformed how healthcare professionals respond to emergencies, ensuring the safety of patients in emergencies is still paramount.

This Special Issue entitled “Emergency Medicine: Clinical Advances and Challenges in Diagnosis and Treatment” has attracted 13 high-quality articles contributed by authors from six different countries: Poland, Korea, Taiwan, Romania, Germany, and Italy. These articles highlight the breadth and depth of research in the field, offering insights into the latest advancements and personalized approaches.

## 2. An Overview of Published Articles

The two studies conducted by Rakesh J. et al. (Contributions 1 and 2) aimed to investigate the usefulness of serum S100B protein levels as a short- and long-term prognostic factor in patients with ischemic stroke and [1] the diagnostic values of S100B in brain damage related to TBI (traumatic brain injury), pointing out that the pivotal role of this protein obtained using these data indicates that S100B may be regarded as a therapeutic target for acute brain injury. Serum biomarker S100B has been explored for its potential role in improving clinical decision-making in the management of patients suffering from ischemic stroke [2]. 

The detection of high S100B levels in peripheral circulation after acute ischemic stroke and the correlations of S100B levels with infarct size (good) and disability (poor) imply that S100B protein may be used as a peripheral marker in acute ischemic stroke patients. To reduce costs and ED overcrowding and minimize radiation exposure, biomarkers are urgently needed to find patients for whom a CT scan can be avoided. S100B is the most studied astroglia and blood–brain barrier biomarker in traumatic brain injury (TBI). The serum levels of S100B in patients with TBI indicate patient outcomes, where elevated levels correlate with injury severity and mortality. In a parallel effort, authors have initiated the proposal of criteria that utilize serum S100B analysis in the TBI diagnostic process based on NICE guidelines and highlighted the need for more extensive multicenter studies to establish its integration into the clinical algorithm as S100B protein proves to be a promising diagnostic tool [1].

Emergency departments (ED) place a high priority on perfecting the management of acute ischemic stroke. The time-sensitive nature of intervention for stroke patients has been emphasized, with guidelines suggesting the administration of intravenous (IV) recombinant tissue plasminogen activator (rt-PA) within 4.5 h of symptom onset and the initiation of endovascular procedures “as early as possible” when necessary. According to a study conducted by Daian Popa et al. (Contribution 3), there is potential for improvement in the thrombolysis rate at Municipal Clinical Hospital Timișoara, Romania, despite being relatively favorable compared to global benchmarks. The research revealed a noteworthy association between the highest door-to-CT time and mortality rates for all patients, emphasizing the importance of meeting emergency department (ED) time targets. The findings strongly recommend that hospitals lacking a resolute stroke unit reorganize their acute ischemic stroke (AIS) management protocols to prioritize achieving ED time targets. By doing so, hospitals can enhance their response to AIS cases, ultimately influencing the outcomes of AIS patients and facilitating smoother inter-hospital transfers [3].

Hyelin H. et al. (Contribution 4) focused on developing and confirming a simple tool for predicting bacteremia, a scoring system that helps classify bacteremia risk, stratify patients earlier, and initiate prompt treatment in high-risk patients. Scoring systems can supplement physicians’ clinical judgment when deciding whether to perform a blood culture and start treatment. In their retrospective cohort study, conducted over five years, they developed and confirmed a simple bacteremia prediction score. It used only five variables and proved a similar performance to the model with sixteen variables using all laboratory results and vital signs [4].

Antimicrobials are one of the most prescribed drug classes in the ED. The global increase in antimicrobial resistance (AMR) is one of the leading international health threats, reducing the effective treatment of infections, increasing the need for intensive care units (ICUs), the length of stay, healthcare costs, and significantly affecting patient morbidity and mortality. Pathogen resistance develops in response to selective pressure associated with all antibiotic prescribing but is accelerated by inappropriate use. Musuroi S. et al., (Contribution 5) in their retrospective observational study, aimed to evaluate the etiology and antimicrobial resistance (AMR) pattern of community-acquired pathogens, as well as the epidemiological characteristics of patients admitted through the ED, to guide appropriate antibiotic therapy and underlined the imperative introduction of rapid microbiological diagnostic methods in ED to identify AMR strains and improve therapeutic protocols [5].

Hip fracture injuries have been identified as one of the most serious healthcare problems, associated with a high rate of mortality and profound temporary or permanent impairment of quality of life. The effect of delayed surgery on postoperative outcomes has been widely discussed. The demand for early surgery often exceeds available resources, and considerable variability among hospitals in time to surgery has been reported. Confirmation of the clinical consequences of surgical delay on different patient groups could help in the decision-making process when not all patients can be performed on as early as desired. The optimal time for surgery associated with the lowest risk of complications after hip fracture surgery is still under debate. The most controversial factor recognized and examined in multiple studies of hip fractures is the “time-to-surgery” interval, which includes also the time in the emergency department (ED). Authors Daginnus et al. (Contribution 6) analyzed in a study over five years whether the occurrence of complications after the treatment of hip fractures differs according to the patient’s type of fractures and the specific treatment procedures. The authors concluded that a 24 h time interval between injury and surgical procedure plays a significant role in extracapsular fractures treated with osteosynthesis but not in intracapsular fractures treated with arthroplasty. Therefore, they recommend reevaluating guidelines on hip fracture surgery according to the patient’s case scenario, particularly the individual hip fracture type [6].

Imaging plays a significant role in assessing pulmonary diseases and currently serves as a tool to diagnose lung pathology, check its course, and guide clinical management. Lung ultrasound is a real-time imaging modality that is non-invasive, simple, and free of ionizing radiation. Due to unique features and growing scientific evidence, lung ultrasound (LUS) is an emerging technique for bedside chest imaging in critical care. While the utility of lung ultrasound in the emergency setting is unquestioned, its potential role in the more complex and resource-rich intensive care environment is still under debate. The purpose of this paper by Marco Di Serafino et al. (Contribution 7) is to underline the role of point-of-care LUS in ICUs from a purely radiological point of view as an advanced method in ICU CXR reports to improve the interpretation and monitoring of lung CXR findings [7].

In a study over nine years and a literature review, authors Faur C et al. (Contribution 8) highlighted the importance of early diagnosis and efficient management of axillary arterial injuries associated with proximal humeral fractures, emphasizing the importance of heightened clinical awareness to avoid oversight for these less common injuries. Axillary artery injury due to proximal humerus fracture is rare, although its incidence is uncertain. As the number of inpatient admissions for proximal humerus fractures is on the rise, we are now detecting more axillary artery injuries than we did in the past. Recognition at first presentation can sometimes be complex owing to palpable peripheral pulses and the absence of ischemia. Increased awareness of this injury should be maintained, mainly when paresthesia and concomitant injuries (e.g., brachial plexus or scapula fractures) are present. Accurate physical examination, in combination with a low threshold for Doppler examination or angiography, can set up the diagnosis of axillary artery injury [8].

Authors Anglitoiu B. et al. (Contribution 9) presented a case study detailing the successful staged treatment of posttraumatic tibial osteomyelitis using a unique combination of rib graft and serratus anterior muscle, focusing on wound management, infection control, and limb salvage. The tibia is the most common site of posttraumatic osteomyelitis. In recent years, posttraumatic osteomyelitis has become one of the most essential types of exogenous bone infections. This case report proves that with a careful and comprehensive strategy, patients can achieve positive outcomes despite complex and multifaceted clinical challenges. As the field of orthopedic and trauma surgery continues to progress, the lessons from this case serve as a reminder of the potential for innovation and improvement in patient care, particularly for those facing the daunting prospect of posttraumatic tibial osteomyelitis [9].

Cryptococcemia is a rare, life-threatening fungal infection that can occur in immunocompromised patients. It often presents a diagnostic challenge, given its non-specific clinical manifestation that can mimic other diseases. Cryptococcemia requires early identification and prompt antifungal therapy. Authors Wei-Kai Liao et al. (Contribution 10) presented the first study of applying scoring systems in predicting the mortality risk of patients with cryptococcosis and finding higher scores associated with a significantly higher mortality rate in patients with cryptococcosis. The study offers valuable insights for future guidelines in this area [10].

The COVID-19 pandemic has shown vulnerabilities and brought considerable challenges to medical systems, especially Emergency Medicine. Healthcare personnel need regular, up-to-date information and communication to be protected from chronic stress and poor mental health so they can have a better ability to fulfill their roles. The research of Goniewicz et al. (Contribution 11) was performed online from 2021 to 2022 and included medical professionals from diverse healthcare settings, with the survey link in four provinces of Poland. The study highlighted the importance of adaptive, agile, compassionate, and supportive organizational structures, especially during global health emergencies. While many aspects of the pandemic crisis are still being researched, healthcare organizations must be on the frontline. They must play vital functions by updating and following strict infection prevention and control practices. Smart, targeted investments in health system resilience are needed to improve health and ensure the next shock is less disruptive and costly [11].

The retrospective analysis conducted by Marza A. et al. (Contribution 12) offers exciting insights into the uncommon clinical manifestations, such as spontaneous pneumomediastinum (SPM) and spontaneous pneumothorax (SP) associated with COVID-19, from 2020 to 2022 across five pandemic waves, highlighting the importance of early recognition and management of each SPM/SP case. As COVID-19 has become one of the world’s deadliest pandemics known in history, new knowledge has been gained about the virus and its possible complications. The main finding of this analysis shows that the risks associated with mortality, mechanical ventilation, and ICU admission in patients with SP-SPM are greatly influenced by the presence of COVID-19, extensive lung damage, and a higher number of comorbidities. This holds regardless of patient age and the severity of different strains during a pandemic outbreak [12,13].

Mortality prediction in critically ill patients with cardiogenic shock can guide triage and selection of potentially high-risk treatment options. Authors Jen-Wen Ma et al. (Contribution 13) developed a prediction model to assess the mortality risk and provide guidance on treatment for patients with cardiogenic shock based on four parameters: platelet counts, left ventricular ejection fraction, age, and lactate (PEAL). The study between 2014 and 2019 focused on patients with cardiogenic shock in the emergency department, where clinical outcomes and risk factors for 30-day mortality were evaluated. The model is the first risk score incorporating the number of platelet counts at presentation and showed good predictive performance for all-cause mortality at 30 days in all patients. This score can assess the impact of treatment strategies on expected mortality, enable the design of future clinical trials, and serve as a model for developing future risk scores in cardiology [14].

To conclude, the published papers in this Special Issue covered a wide range of topics. We want to express our gratitude to these authors for their invaluable contributions and dedicated efforts to research. The knowledge and perspectives they have shared have the incredible ability to influence the trajectory of Emergency Medicine, propelling the concept of tailored healthcare. We urge readers to explore the articles and embrace the revolutionary impact of groundbreaking interventions as they strive to elevate the quality of patient care.
